# Two-Year Longitudinal Analysis of a Cluster Randomized Trial of Physical Activity Promotion by General Practitioners

**DOI:** 10.1371/journal.pone.0018363

**Published:** 2011-03-29

**Authors:** Gonzalo Grandes, Alvaro Sanchez, Imanol Montoya, Ricardo Ortega Sanchez-Pinilla, Jesús Torcal, for the PEPAF Group

**Affiliations:** 1 Primary Care Research Unit of Bizkaia, Basque Healthcare Service (BHS), Bilbao, Spain; 2 Santa Barbara Primary Care Centre, Castilla-La Mancha Healthcare Service, Toledo, Spain; 3 Basauri-Ariz Primary Care Centre, Basque Healthcare Service, Basauri, Spain; Universidad Peruana Cayetano Heredia, Peru

## Abstract

**Background:**

We evaluate the effectiveness of a physical activity promotion programme carried out by general practitioners with inactive patients in routine care.

**Methods and Findings:**

Pragmatic, cluster randomised clinical trial conducted in eleven public primary care centres in Spain. Fifty-six general practitioners (GPs) were randomly assigned to intervention (29) or standard care (27) groups. They assessed the physical activity level of a systematic sample of patients in routine practice and recruited 4317 individuals (2248 intervention and 2069 control) who did not meet minimum physical activity recommendations. Intervention GPs provided advice to all patients and a physical activity prescription to the subgroup attending an additional appointment (30%). A third of these prescriptions were opportunistically repeated. Control GPs provided standard care. Primary outcome measure was the change in self-reported physical activity from baseline to six, 12 and 24 months. Secondary outcomes included cardiorespiratory fitness and health-related quality of life.

A total of 3691 patients (85%) were included in the longitudinal analysis and overall trends over the whole 24 month follow-up were significantly better in the intervention group (p<0.01). The greatest differences with the control group were observed at six months (adjusted difference 1.7 MET*hr/wk [95% CI, 0.8 to 2.6], 25 min/wk [95% CI, 11.3 to 38.4], and a 5.3% higher percentage of patients meeting minimum recommendations [95% CI: 2.1% to 8.8%] NNT = 19). These differences were not statistically significant at 12 and 24 months. No differences were found in secondary outcomes. A significant difference was maintained until 24 months in the proportion of patients achieving minimum recommendation in the subgroup that received a repeat prescription (adjusted difference 10.2%, 95% CI 1.5% to 19.4%).

**Conclusions:**

General practitioners are effective at increasing the level of physical activity among their inactive patients during the initial six-months of an intervention but this effect wears off at 12 and 24 months. Only in the subgroup of patients receiving repeat prescriptions of physical activity is the effect maintained in long-term.

**Trial Registration:**

clinicaltrials.gov NCT00131079

## Introduction

The benefits of physical activity for health promotion and prevention of most common chronic diseases are so great that it is probably the most important healthy habit to maintain and the most useful self help treatment available. Regular physical activity improves quality of life, prevents cardiovascular and respiratory disease, obesity, type 2 diabetes, hypertension, osteoporosis, some cancers, and depression, improves the symptoms of anxiety and other illnesses, and decreases all-cause mortality [1]–[2]. Accordingly, public health authorities and scientific organizations recommend at least 30 minutes of moderately intense physical activity most days of the week [1], [3]. Despite this, inactive and sedentary lifestyles remain a problem for the majority of the population in industrialized countries, overall levels of physical activity continue to be low or are even declining [1], [4], [5], and physical activity promotion represents a public health priority.

General practitioners can play a key role in population health throughout physical activity promotion. However, evidence about these interventions in routine practice has so far been inconclusive, especially regarding their long-term effectiveness [6]–[7]. This uncertainty generates disagreement between scientific organizations and public health agencies on what general practitioners should do. While some organisations recommended that primary care practitioners should take the opportunity, whenever possible, to identify inactive adults and advise them [8], other conclude that the evidence is insufficient to recommend for or against behavioural counselling in primary care [9]. The purpose of our study is to address such a relevant question: which is the specific effect general practitioners have when they try to promote physical activity among their inactive patients.

We recently reported general practitioners to be effective at promoting physical activity for a six-month period [10]. However, maintenance of an active lifestyle is essential to achieve health benefits and evidence for the long-term effectiveness of interventions is urgently required [6], [7]. There are few clinical trials in the primary care setting with positive long-term results beyond 12 months [6], [7]. B. A. Lawton et al found that the “green prescription” intervention can increase physical activity of inactive women over two years. This intervention included multiple sessions with a primary care nurse and a community exercise specialist over a nine-month period. The effect of the intervention on physical activity levels increased for as long as 12 months, with a further decline at the end of two years. The authors acknowledged the limited generalizability of their results due to the biased selection of participants and because the intervention was not part of routine care [11]. The Activity Counseling Trial compared three different intensity interventions over two years and found no effects on physical activity. The two more intensive interventions produced a greater improvement in cardiorespiratory fitness in the subgroup of women. However, this study had no control group and therefore it did not directly address the central question considered in the present study that remains unanswered, namely, whether family physicians' interventions in routine conditions increase their patients' physical activity in the long term [12], [13].

In the reported preliminary results of this clinical trial, the PEPAF (“Experimental Programme for Physical Activity Promotion”) programme implemented by general practitioners in routine practice was shown to be effective at six months follow-up [10]. As the PEPAF programme is a brief intervention based on physicians' behavioural counselling and prescription, it is expected that some subjects that increased physical activity at six months might fall back into their previous routine of inactivity. We report the final results on the long term effectiveness of the PEPAF programme to increase physical activity of primary care inactive patients throughout 24 months after the beginning of the clinical trial.

## Methods

The PEPAF project was a pragmatic cluster randomized controlled clinical trial initiated in October 2003 at 11 primary care centres in Spain [10]. The present study reports the long-term effectiveness at six, 12- and 24-month follow-up measurements, performed through March 2006.

### Ethics Statement

The study complies with the guidelines of the Declaration of Helsinki. The study was approved by the Clinical Research Ethics Committees of the participating centres.

### Participants and randomization

**Figure 1:**
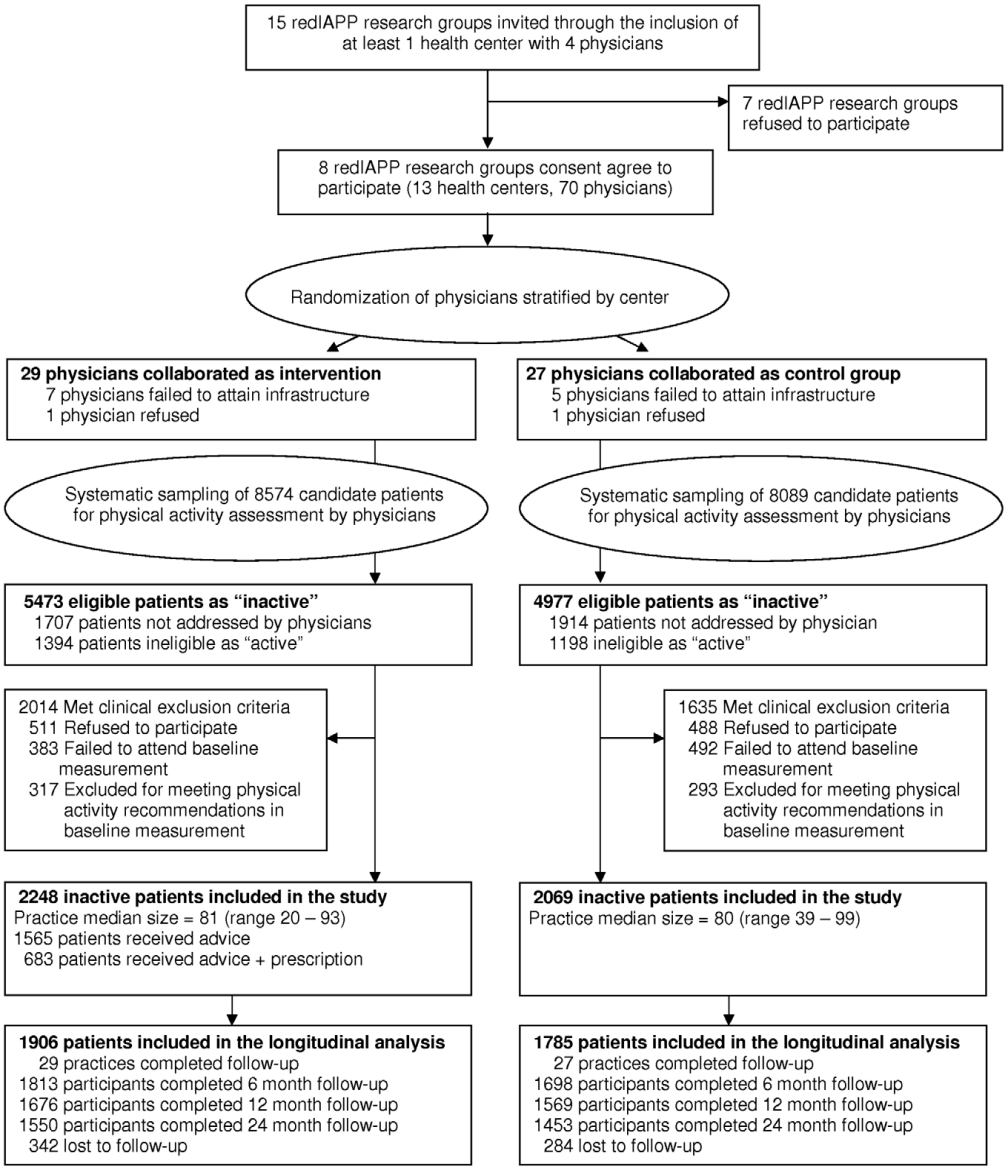
Flow chart of the PEPAF trial.

General practitioners were invited to participate through the Spanish Preventive Services and Health Promotion Primary Care Research Network. A detailed description of study setting and participant recruitment is given elsewhere [10], [14]. In short, a total of 70 family physicians as allocation units from 13 health centres were randomized before patient recruitment, in a 1∶1 ratio using computer-generated random numbers provided by a central site, the Primary Care Research Unit of Bizkaia. Randomisation of physicians was stratified by centre. Two health centres (12 physicians) dropped out before the start of the study due to technical difficulties, and two practices refused to participate. In the end, 56 GPs from 11 primary health care centres initiated and completed the study, 29 allocated to the PEPAF group and 27 to the control group (Figure 1).

A sample of 16663 patients aged 20–80 from those scheduled for an appointment during the recruitment period was selected by research nurses using systematic sampling. Physicians, after dealing with the reason for the consultation and guided by web-based software, screened the physical activity level of 13042 selected patients. Due to non-attendance, severity of problems, technical difficulties or lack of time, 3621 selected patients were not assessed. Of those assessed, 2592 were identified as active, while 10450 were eligible for the study (5473 and 4977 allocated to the intervention and control groups, respectively) because they did not meet the recommended aerobic physical activity levels (moderate-intensity physical activity for ≥30 minutes 5 d/wk or vigorous intensity activity for ≥20 minutes 3 d/wk) [3] (Figure 1). The negative answer to the screening questions used by doctors to identify active patients had a predictive value of 87.6% [14].

Computer screen shots guided physicians in reviewing exclusion criteria: cardiovascular disease or other conditions that could preclude exercising safely or could be exacerbated by exercise, severe emotional distress, complicated pregnancies, and follow-up difficulties. In the intervention and control groups, 2014 and 1635 eligible patients, respectively, met some exclusion criterion; 511 and 488, respectively, refused to participate when offered an informed consent form; and 383 and 492, respectively, failed to attend the baseline measurement session. Written informed consent was obtained from all participants involved in the study. Of the 4927 patients who completed the baseline measurement, 317 intervention patients and 293 control patients were excluded because they already met the minimum recommended levels of physical activity, as confirmed in the baseline physical activity assessment performed by research nurses. Finally, a total of 2248 intervention patients and 2069 control patients were included in the study (Figure 1).

### Interventions

Figure S1 shows a graphical depiction and a detailed description of the intervention. Assisted by web-based software, general practitioners allocated to the PEPAF group provided brief advice and educational materials focusing on the benefits of physical activity and the risks of inactivity, and offered an additional 15-minute appointment to prescribe an individualized physical activity plan. Patients attending and not attending this appointment formed the prescription and advice subgroups, respectively. In the case of the prescription subgroup (30% of the intervention patients), after addressing potential barriers to and solutions to enable change, physicians negotiated and prescribed a 3-month physical activity plan aimed at gradual achievement of the recommended physical activity levels (3), that resulted in a standardized printed prescription with a self-monitoring log. Physicians were suggested to review the physical activity plan under an opportunistic strategy, resulting in the repetition of 32.1% of the initially prescribed plans. Quality of the recruitment and intervention delivery processes was ensured by an intensive training of general practitioners and the web-based software, which obliged them to advance through several screens containing standardized contents of the intervention, and registered the process for each patient. Control group physicians assessed patients' physical activity and performed recruitment in a similar way to that of the intervention group ones, but they delayed any systematic intervention related to physical activity until the end of the study, unless the reason for consultation or the patients' health problems were directly related to inactivity. Further description of intervention content, its theoretical base, physicians' training and standardization procedures is given elsewhere [10], [14]. The protocol for this trial and the CONSORT checklist are available as supporting information; see Protocol S1 and Checklist S1.

### Measurements and follow-up

The primary outcome measure was the change in physical activity level estimated using the *7-day Physical Activity Recall (7-day PAR)* semi-structured interview [15]. The *7-day PAR* records the time spent on all kinds of leisure and occupational activities lasting more than 10 accumulated minutes in the 7 days prior to the interview. Minutes per week spent doing moderate and vigorous physical activity and the proportion of participants who achieve the minimum recommended physical activity levels are directly calculated, while weekly activity dose in metabolic equivalents - hour per week (MET*h/week) is estimated by multiplying the hours devoted to activities of moderate, hard, and very hard intensity by the corresponding METs: 4, 6, and 10 respectively. The *7-day PAR* validity has been accredited many times over the years, including with Hispanic populations [16], and we evaluated it in a sample of 160 participants in our study, obtaining good reliability indexes (intraclass correlation>0.6) and a correlation of 0.52 with an objective measurement of activity dose (accelerometer).

Secondary outcome measures included maximal oxygen uptake indirectly estimated by the YMCA cycle ergometer submaximal exercise test [17] and health-related quality of life using the Spanish version of the SF-36 questionnaire (version 1) [18]. We consider as possible confounders baseline motivational stage of change, assessed by a self-administered questionnaire [19]; social class and educational level [20]; excessive alcohol consumption, using the Spanish version of the AUDIT (risky drinker ≥8 points) [21]; smoking status and demographic variables reported by patients; and risk factors extracted from patients' clinical records.

Trained nurses working in exercise laboratories who performed baseline and follow-up measurements at 6, 12 and 24 months were blinded to the allocation group of the participants. The quality of measurements was assured by training of research nurses, a pilot study, and double data entry into a central Oracle™ database. A telephone recall system was used to improve follow-up rates of patient measurements. Quality control was performed by the coordinating center (Primary Care Research Unit of Bizkaia) with daily online supervision and feedback to nurses regarding the study process and data entry, monthly progress reports, and regular meetings every four months with the collaborating investigators and research nurses.

### Statistical and power analyses

To test overall effect of the intervention on an intention-to-treat basis, we compared changes in outcome variables between the two groups over the three follow-up measurements adjusted by baseline values. Longitudinal generalized mixed models were used to take into account the repeated measurements for each patient and also the hierarchical structure of data, with patients nested in doctors and health centers (SAS PROC MIXED and GLIMMIX ver. 9.2, SAS Institute, Cary, NC, USA, 2009; SAS code available upon request). These models were linear for changes in physical activity, VO2max, and quality of life and logistic for achievement of the minimum recommended physical activity levels. Time evolution was considered in these models as a continuous variable, based on linear, quadratic or logarithmic functions or as a categorical variable, with several correlated measurements for each individual. This last option was chosen since it is the less restrictive and provided a better fit to our data. The PEPAF intervention, the time of measurement and intervention-by-time interaction were included as fixed effects in the models. Patients, doctors and centres were included as random effects in the intercept and in the slope of the different repeated measurements. These models were also adjusted for baseline values of the outcome variables, the season of the year in which the measurements were made, known determinants of physical activity and physician characteristics. Different covariance structures were used for the repeated observations on the same patient, doctor and centre and restricted maximum likelihood ratio tests were used to determine the best covariance structure for our data. Likewise, to simplify the fixed effects structure, maximum likelihood ratio tests were used following backward, forward and stepwise strategies (significance criterion p<0.05).

We assessed the overall effect of the PEPAF programme by testing the interaction between intervention and time of measurement. When this intervention by time interaction was significant (significance criteria p<0.05), planned contrasts were used to determine whether changes in the PEPAF group between baseline and each of the follow-up points were significantly different from those observed in the control group (p<0.05). Finally, to describe the effect of prescription in addition to advice, a predefined per-protocol analysis was undertaken testing the “intervention group by prescription” interaction (p<0.01).

In addition to previously mentioned random structure, random effects on the PEPAF programme effect at centre level were included to test whether the effect attributable to the intervention varied across centres. Empirical Bayesian estimators were calculated for each centre, followed by a sensitivity analysis to evaluate changes after excluding centres whose populations significantly differed from the overall average. Given the skewness of the continuous outcome variables, sensitivity analysis was repeated excluding patients considered as potential outliers, namely, those beyond two standard deviations. No imputation method was used to handle the missing data since longitudinal mixed models based on maximum likelihood estimation used in this article are more appropriate to deal with missing data [22] than common imputation methods such as last observation carried forward, complete case analysis or other possible forms of imputation.

A post hoc power calculation based on longitudinal mixed-effects models adjusted to the final sample size, actual data variability, and clustering showed that the study has a power greater than 95% to detect a minimal difference between comparison groups of at least 2.25 METh/wk, 0.80 mL/kg/min in VO2max and 5 points in SF-36 health-related quality of life scores at any measurement point.

## Results

A total of 3691 patients with at least one follow-up measurement (85% of the 4317 recruited in the study) were included in the longitudinal analysis, 1906 from the PEPAF group and 1785 from the control group (Figure 1). Baseline characteristics of these participants were balanced between both comparison groups with regard to the primary and secondary outcomes, as well as to the majority of sociodemographic and risk factors. The mean age was 50.6, 64.6% were women, the average time devoted to moderate or vigorous activities was 34.7 min/wk and the mean weekly activity level was 2.4 MET-h/wk. The control group had a higher proportion of patients with dyslipidemia (p-value<0.001), and with low levels of education (p-value = 0.003), and a lower proportion in the “preparation” stage of change (p-value<0.001). Additionally, baseline, 12 and 24-month measurements were mostly made in autumn and winter, whereas the majority of 6-month measurements were performed in spring and summer. To control for these differences, subsequent analyses were adjusted for these variables (see TABLE 1).

**Table 1:** Baseline characteristics of the 3691 primary care patients included in the longitudinal analysis.

	Intervention Group	Control Group
	(N = 1906)	(N = 1785)
Primary outcomes		
Physical activity dose, METs * hour/week	2.46 ( 6.08)	2.37 ( 5.88)
Moderate and vigorous activity, minutes/week	35.94 (94.81)	33.45 (77.81)
Secondary outcomes		
VO_2_max, ml/kg/min^a^	24.30 (8.12)	24.63 (8.36)
Physical Component Summary^b^	48.11 (8.26)	47.70 (8.39)
Mental Component Summary^b^	45.87 (11.71)	46.76 (11.51)
Sociodemographic variables		
Age, y	50.28 (14.66)	51.01 (14.80)
Female *n (%)*	1259 (66.0)	1125 (63.0)
Work Situation *n (%)*		
Works outside of home	956 (50.6)	882 (49.4)
Homemaker	469 (24.6)	424 (23.7)
Retired	283 (14.8)	325 (18.2)
Student	46 (2.4)	30 (1.7)
Unemployed	91 (4.8)	78 (4.4)
Other	52 (2.7)	46 (2.6)
Educational level *n (%)*		
None	82 (4.3)	137 (7.7)
Elementary School	592 (31.1)	546 (30.6)
Middle or High School	894 (46.9)	812 (45.5)
University studies	388 (17.7)	290 (16.2)
Social Class^c^ *n (%)*		
Manager large enterprise	126 (6.6)	130 (7.3)
Manager small enterprise	223 (11.7)	180 (10.1)
intermediate employee	551 (28.9)	541 (30.3)
Manual worker	1006 (51.9)	934 (52.3)
Risk factors *n (%)*		
Diabetes	145 (7.6)	163 (9.1)
Hypertension	466 (24.4)	473 (26.5)
Dyslipidemia	381 (20.0)	429 (24.0)
Obese (≥30 kg/m^2^)	479 (25.1)	455 (25.5)
Current smoker	575 (30.2)	506 (28.3)
At-risk drinker *n (%)*	104 (5.5)	83 (4.7)
Physical activity stage of change *n (%)*		
Pre-contemplation	396 (20.8)	640 (35.8)
Contemplation	629 (33.0)	611 (34.23)
Preparation	617 (32.4)	319 (17.9)
Action	92 (4.8)	55 (3.1)
Maintenance	172 (9.0)	160 (9.0)

Values are means (standard deviation) unless otherwise stated.

a Variables derived from the cycle ergometer test at baseline: n = 3089.

b Sample size for Physical and Mental Component Summary: n = 3652.

c Social class classification based on occupation and work position (30): Class IV to V includes non-qualified and qualified manual workers; Class III includes the administrative workforce, supervisors and freelance workers; Class II includes managers of enterprises with less than ten employees, professionals with first level university degree, senior technicians, artists and sportsmen/women; Class I includes managers of public organizations or private enterprises with more than ten employees, professionals with second and third level university degrees.

**Figure 2:**
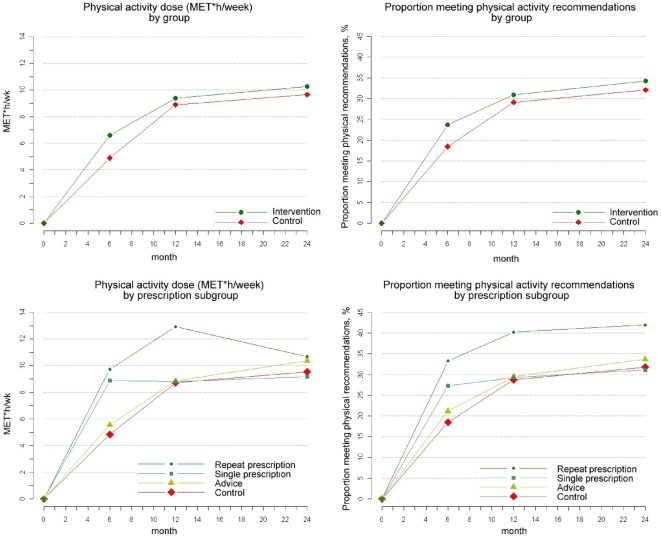
Two years longitudinal change in physical activity by comparison groups and subgroups.

Both groups increased activity levels over the 24 month follow-up period and the overall evolution was more favourable in the intervention group (p<0.01) which showed higher increments in physical activity at every follow-up point (TABLE 2, Figure 2). At six months, compared with controls, those included in the PEPAF group increased physical activity by 1.72 more MET hours/wk (95% CI: 0.79 to 2.65), by 25 more min/wk devoted to moderate or vigorous activities (95% CI: 11.3 to 38.4) and by a 5.3% more of the participants meeting the minimum physical activity recommendations (95% CI: 2.1% to 8.8%). At the 12- and 24-month follow-ups these differences, although still favourable to the intervention group, were lower and not significant (p>0.05) (TABLE 2, Figure 2). Age, sex and baseline stage of change, did not modify the effect of the PEPAF intervention on the longitudinal 24-month evolution of physical activity changes. The secondary outcome analysis showed a small but significant improvement in both groups in their VO2max levels and health-related quality of life scores over time. However, no significant differences were found between groups in the secondary outcomes (p>0.05). (Table 2)

**Table 2:** Longitudinal changes in primary outcomes: physical activity level.

	Baseline adjusted change (95% CI)		Multivariate-adjusted attributable difference (95% CI)	Intervention -time of measurement interaction
	Intervention Group	Control Group		
	(n = 1906)	(n = 1785)		p-value
**Primary Outcomes**				
Physical activity dose, MET-h/wk				0.004
6 months	7.07 (4.85–9.29)	5.32 (3.10–7.54)	1.72 (0.79–2.65)	
12 months	7.76 (5.51–10.00)	7.29 (5.04–9.54)	0.49 (−0.60–1.59)	
24 months	9.09 (6.81–11.37)	8.29 (6.00–10.58)	0.62 (−0.62–1.86)	
Moderate and vigorous activity, min/wk				0.003
6 months	119.15 (86.50–151.80)	95.06 (62.36–127.76)	24.87 (11.33–38.40)	
12 months	128.60 (95.73–161.47)	127.33 (94.40–160.26)	2.01 (−12.56–16.59)	
24 months	148.82 (115.41–182.23)	139.97 (106.49–173.46)	7.33 (−9.75–24.42)	
Proportion meeting physical activity recommendations, %				0.009
6 months	22.36 (17.56–28.03)	17.46 (13.47–22.34)	5.27 (2.08–8.79)	
12 months	23.46 (18.38–29.45)	21.92 (17.05–27.70)	1.80 (−1.97–5.85)	
24 months	27.84 (21.09–35.78)	25.69 (19.27–33.37)	2.14 (−1.81–6.32)	
**Secondary Outcomes**				
Estimated maximal oxygen uptake: VO2 mL/kg/min^a^				0.368
6 months	1.62 (1.10–2.14)	1.41 (0.88–1.94)	0.24 (−0.14–0.61)	
12 months	1.10 (0.56–1.64)	1.21 (0.66–1..75)	−0.11 (−0.53–0.31)	
24 months	1.06 (0.51–1.62)	0.86 (0.30–1.42)	0.18 (−0.28–0.64)	
Health Related Quality of Life: SF-36 Physical Component Summary^b^				0.954
6 months	1.43 (0.64–2.23)	1.47 (0.67–2.27)	−0.05 (−0.48–0.38 )	
12 months	1.20 (0.40–2.01)	1.30 (0.49–2.11)	−0.10 (−0.58–0.38 )	
24 months	1.26 ( 0.45–2.08)	1.20 (0.39–2.02)	−0.06 (−0.44–0.55 )	
Health Related Quality of Life: SF-36 Mental Component Summary^b^				0.510
6 months	0.92 (−0.34–2.18)	0.67(−0.59–1.93)	0.43 (−0.15–1.02)	
12 months	1.18 (−0.09–2.44)	1.30 (0.03–2.56)	0.06 (−0.55–0.67)	
24 months	1.64 (0.38–2.91)	1.85 (0.58–3.12)	0.00 (−0.63–0.63)	

Intention-to-Treat Analysis.

a Sample size for the estimated maximal oxygen uptake n = 3089 (Intervention group n = 1598, control group n = 1491).

b Sample size for Physical and Mental Component Summaries: n = 3652 (Intervention group n = 1886, control group n = 1766).

When analyzing the treatment actually received within the programme, the trend to physical activity gain only appeared in the prescription subgroups while the advice subgroup showed non significant differences with the control group at every follow-up point (PEPAF intervention by prescription subgroup interaction p-value<0.007, see figure 2). At six months follow-up, those receiving a prescription were 100% more active than controls, with a difference of 4.17 METS hour/wk (95 CI% 2.80 to 5.54). At 12 months this difference disappeared for the subgroup receiving a single prescription but remained for those who repeated the prescription (difference of 4.21 METS hour/wk; 95% CI 1.68 to 6.74). At 24 month follow-up the prescription effect on activity dose was not significant (1.13 METS hour/w, 95% CI −1.74 to 4.01). However, in terms of the proportion of participants meeting the minimum physical activity recommendations, the effect of the repeat prescription remained significant until the end of 24 months follow-up. For those having received a repeat prescription this percentage surpassed that of the control group by 14.83% at 6 months (95%CI 7.38% to 23.21%), by 11.49% (95%CI 3.08% to 20.51%) at 12 months, and by 10.17% (95% CI 1.48% to 19.36%) at the 24 month follow-up (Figure 2). Baseline characteristics of the patients who received a single prescription and of those who received a repeat prescription were balanced in most variables. However, among those patients who received a single prescription there was a higher proportion of obesity and their mean mental health component score was lower than those who received a repeat prescription.

While there was a significant variability in changes in physical activity between the populations from the different collaborating centres at every follow-up point (p-value <0.001), the within-centre effect attributable to the intervention did not vary across centres (p-value >0.44). The seasonal effect was significant in all the analyses (p-value <0.0001). For the multivariate multilevel model of the change in physical activity the estimated effect of the season of the year in which measurements were made was the results being higher by 3.65 METs*hour/week for those measured in the spring, 1.89 for those from the summer, and 1.80 for the autumn compared to those measured in the winter. Finally, the sensitivity analysis excluding populations from centres that significantly differed from the average showed no relevant changes in either the direction or the magnitude of the observed effects. When excluding patients considered as outliers, the overall evolution was more favourable to the PEPAF programme because not only was the intervention effect significant at 6 months, it also remained significant until 12 month follow-up in METs hour/week and minutes of moderate or vigorous activity (p<0.05).

## Discussion

Physical activity increased significantly more for primary care patients exposed to the PEPAF programme than for patients in the control group. This improvement was shown during the initial six-month follow-up, but the effect of the programme declined and lost significance over 12 to 24 months. The mean effect size achieved at six months may be considered of moderate clinical relevance at an individual level, but a 5.3% higher percentage of patients achieved the minimum recommended physical activity level in the intervention group, this being relevant for public health. After this six-month period, the effect attributable to the intervention was lost. This trend of the effect of interventions diminishing in the longer term after an initial increase has been described in previous studies [7], [11].

A loss of effectiveness in the very long term is expected as a reflection of the lack of a continuous or ongoing intervention, clinical reinforcement strategies and support in the community. Indeed, comprehensive interventions combining informational, behavioural and environmental approaches can be more effective in the long term than interventions delivered only by physicians [7]. The available evidence on interventions in the primary care setting with 24 months of follow-up is mainly restricted to two high quality studies that used the following intervention components in addition to advice and information material: first, written prescriptions of exercise by nurses or doctors linked to community-based exercise facilitators or health educators who offer support for goal setting, tailoring of physical activity plans, and strategies for overcoming barriers; second, booster and reminder strategies via telephone, internet, mail, personal meetings, follow-up workshops, group sessions, printed materials or newsletters; and third, feedback to the health professionals and updating of the physical activity prescriptions [11], [12], [23]. However, evidence remains scarce with respect to which components of such interventions are effective and efficient in the long term, and this warrants further investigation [7].

When analyzing the treatment actually received by participants within the programme, a trend toward very long-term effectiveness is associated with the repeat prescription subgroup. Those participants who had repeated the individual physical activity prescription showed clinically relevant differences at 12 months in weekly activity levels with respect to the control group and even at 24 months in the proportion meeting the physical activity recommendations. The interpretation of these on-treatment analyses which lose the strength of randomization must be very cautious. When attributing results to the physical activity prescription, even after multivariate adjustment, residual confounding due to the self-selected nature of these subgroups may be present. However, this analysis illustrates what might be expected for patients who accept and repeat the prescription of a physical activity plan. The long-term sustainability of the results in this subgroup is consistent with the booster effect of interventions which provide people with professional guidance in starting an exercise programme and then provide ongoing support [6].

Long term increases in physical activity were associated with improvements in cardiorespiratory fitness but these did not translate into between-group differences, probably because most patients increased physical activity through moderate intensity activities. While the Activity Counseling Trial found significant improvements in cardiorespiratory fitness in the subgroup of women allocated two more intensive interventions [12], no differences associated to physical activity prescription were observed in our subgroup analysis. In any case, when interpreting these results it should be taken into account that physical activity *per se* has an influence on health, one that is not mediated by an increase in fitness or by an improvement in the risk factor profile [24]. No effect on quality of life was observed, which is consistent with results in previous studies [25]. Adverse effects were not considered because there is no evidence that physical activity interventions cause harm [6].

### Strengths and limitations

Comparing this study with others previously published, the recruitment of a larger unselected sample of inactive patients, not especially motivated to change, and the two-year follow-up give our results greater generalizability and the power to enable results to be analyzed in small subgroups. Overall, the patient characteristics are representative of the common sociodemographic and clinical characteristics seen in primary care [14], and the intervention was performed in routine primary care conditions. A significant variability between physical activity changes seen in the populations from the different centres, along with homogeneity of the intervention effect across centres and their null influence in the sensitivity analysis, supports the generalizability of the results to other similar public primary care populations. The longitudinal analyses used in this article have advantages over the cross-sectional ones that we previously reported concerning the preliminary results of the PEPAF clinical trial at 6-month time point [10]. Longitudinal mixed models used in this article are more appropriate to deal with missing data and standard errors for intervention effects at each time point are calculated using information from all the three measurement points and are therefore more robust than those calculated in our previous paper.

The main limitation of the study is the self-reported measurement of physical activity, which may be associated with recall and social desirability bias. Although it would have been more valid to use objective measures of physical activity, this would have been impractical in such a large scale study with thousands of patients and multiple measurements. Structured self-reported measurements are an accepted method in population-based and clinical studies, and this has been the method used in epidemiological studies linking physical activity and health. In particular, the 7-day Physical Activity Recall has been shown to correlate well with objective measures in previous studies and in a sub-sample of our study population [15], [16], [26], [27]. Another weakness is the lack of blinding of participants. Outcome assessment was blinded to minimize to some extent this bias. Specifically, intervention and control subjects performed the same interview with blinded nurses, and accordingly measurement error is expected to be non-differential. Randomization of physicians before patient recruitment prevented concealment in the patient enrolment process. To minimize a potential recruitment bias, patients to be assessed for inclusion in the study were randomly selected. Despite this measure, it was found that there were a higher proportion of “prepared” patients in the PEPAF group; accordingly, baseline stage of change was considered as a confounder and all analysis were adjusted for this. Since the proportions of people in action and maintenance were similar and physician counselling has been shown to have an immediate effect on patients' readiness to change [28], an explanation, other than recruitment bias, would be the effect of medical advice received by this group immediately before the baseline measurement, causing a transfer of “precontemplators” toward the self-reported “preparation” stage of change.

Another factor contributing to the reduction in the measured effectiveness of the intervention over the longer term in our study is the significant increase in physical activity in the control group, a phenomenon that has also been found in other studies [6], [7], [11], [12]. This might be due to an effect of the repeated physical activity assessment and research procedures implemented by doctors; a seasonal effect resulting from baseline, 12 and 24-month measurements mostly having been made in autumn and winter and 6-month follow-up measurements during spring and summer; and especially, to an effect of the repeated measurement of physical activity, fitness and other examinations and questionnaires, which involved approximately 45 minutes with a research nurse and might have acted as an intervention in itself [29], [30]. This intensive measurement may have given patients an increased awareness of their own level of physical activity and fitness status, resulting in behavioural change. It is also possible that there was contamination of control physicians; randomization by centre would have been more effective to control this. However, it was also important to control heterogeneity and variability from centre to centre; and for this reason it was decided to randomize doctors stratified within centres. As there was some within-centre correlation, this option can be judged to have increased the power of the study and experts have commented that concern about contamination is frequently overemphasized [31].

### Conclusions

General practitioners can enable inactive patients to increase their levels of physical activity, but without reinforcement strategies the results tend to decline over time.

Repeat prescription of a physical activity plan can produce sustained increases in physical activity with great health impact. According to the literature, comprehensive approaches involving repeated interventions that include behaviour change techniques and booster programs might enhance the long-term effectiveness of advice given by physicians and other health care professionals [7]. Yet, the main challenge remains how to integrate these kinds of interventions into routine primary care in a feasible and sustainable way. Future studies should address the modelling and evaluation of these new and complex interventions [32], [33].

## Supporting Information

Figure S1 Graphical depiction of the PEPAF clinical trial.
(PDF)

Checklist S1 (DOC)

Protocol S1 (PDF)
