# Supplementary material for: Two-Year Longitudinal Analysis of a Cluster Randomized Trial of Physical Activity Promotion by General Practitioners
Source: PLoS One. 2011 Mar 29;6(3):e18363. doi: 10.1371/journal.pone.0018363 (PMC3066231; doi:10.1371/journal.pone.0018363)
Supplement: Figure S1 — Graphical depiction of the PEPAF clinical trial. (PDF) [file pone.0018363.s001.pdf]

Graphical depiction of the PEPAF clinical trial

**Timeline**	**Intervention**	**Control**
Research nurse training	(1)	(1)
Assessment visit to collaborating centres	(2)	(2)
Randomization of general practitioners		
Training of general practitioners	(3)	(4)
Recruitment of inactive patients	(5) [a] (6) [b] [c]	(5) [a]
Baseline measurement		
Within one month after recruitment	(7) [d] [e]	
Six months after recruitment	measurement of outcomes	
12 months after recruitment	measurement of outcomes	
24 months after recruitment	measurement of outcomes	

- (1) Three-day training of research nurses in random selection of candidate patients, measurements and data entry. Pilot study and one day review training
- (2) Informed consent to collaborate of at least four practitioners per centre and availability of computer with access to the Internet was required for eligibility
- (3) 3 days x 8 hours course on the theoretical basis of PEPAF and training in intervention strategies for advising, diagnosing patient willingness to change, prescribing physical activity, study protocol, and web-based software
- (4) 5 hours' course on physical activity assessment, study protocol, and training in web-based software to perform recruitment processes
- (5) Physical activity assessment
- [a] Web based software that assisted practitioners to assess physical activity level through a computerized algorithm and further screen-shoots for exclusion criteria review
- (6) Structured physician ADVISE about physical activity benefits and risks of inactivity. Immediately after the advice, practitioners asked patients if they were ready to increase their physical activity level and offered an additional 15 minutes consultation to develop an individualised physical activity plan
- [b] Web based software that prompted open questions, graphical information about risks and standardized sentences to provide medical advice
- [c] A four-page pamphlet summarising the abovementioned information on benefits, risks, motivation, and help offered by general practitioner
- (7) 15 minutes' educational session for the PRESCRIPTION of a physical activity plan, in which general practitioners reinforced patients' reasons and intention to change, and negotiated a goal for patients' physical activity change. They subsequently addressed potential barriers and anticipated solutions for change regarding lack of time, community resources, and health problems. Finally, they designed a 3-month physical activity plan that resulted in a standardised printed prescription that included a self-monitoring log
- [d] Web based software that included standardized sentences, a database with community resources' contact information, evidence-based information for physical activity benefits related to a variety of health problems, and computerized tools to review patients' timetable and identification of free time, and to set goals for physical activity type, frequency, duration, intensity, and progression
- [e] A folder containing a brief guide for increasing physical activity in which the printed prescription was attached
