# Supplementary material for: Two-Year Longitudinal Analysis of a Cluster Randomized Trial of Physical Activity Promotion by General Practitioners
Source: PLoS One. 2011 Mar 29;6(3):e18363. doi: 10.1371/journal.pone.0018363 (PMC3066231; doi:10.1371/journal.pone.0018363)
Supplement: Protocol S1 — (PDF) [file pone.0018363.s003.pdf]

Documento descargado de http://www.elsevier.es el 23/08/2010. Copia para uso personal, se prohíbe la transmisión de este documento por cualquier medio o formato.

Localizador web
Artículo 60.452

Protocolo para la evaluación multicéntrica del Programa Experimental de Promoción de la Actividad Fisica (PEPAF)

G. Grandes[a], A. Sánchez[b], J. Torcal[c], R. Ortega Sánchez-Pinilla[d], K. Lizarraga[e] y J. Serra[e], en representación del grupo PEPAF

Objetivos. Evaluar la efectividad de un innovador programa de promoción de la actividad física (PEPAF) implantado en la consulta diaria del médico de familia.
Diseño. Ensayo clínico controlado y aleatorizado por conglomerados de 100 pacientes, atendidos por cada uno de los 70 médicos colaboradores, asignados a dos grupos paralelos y seguidos durante 24 meses.
Emplazamiento. Trece centros de atención primaria coordinados a través de la Red de Investigación en Actividades Preventivas y de Promoción de la Salud realizadas en Atención Primaria (redIAPP).
Participantes. Muestra probabilística de 7.000 pacientes sedentarios, seleccionada entre todos los que consulten por cualquier motivo a su médico de familia durante el tercer trimestre de 2003. Se excluirán los pacientes con enfermedades cardiovasculares u otros problemas en los que el ejercicio pudiera provocar efectos adversos.
Intervenciones. Los médicos asignados al PEPAF diseñarán un plan de actividad física con los pacientes preparados para el cambio. A los no preparados les aconsejarán brevemente y les entregarán materiales de ayuda. Todos serán objeto de un seguimiento oportunista. Los médicos del grupo control pospondrán cualquier intervención sistemática sobre el ejercicio hasta después de 2005, excepto con los pacientes cuyo problema de salud esté directamente relacionado con el sedentarismo.
Mediciones. La principal medida de resultados será el incremento en el nivel de actividad física, entre la medición basal y la realizada a los 3, 6, 12 y 24 meses, medida con el 7-day Physical Activity Recall. Se medirá también la calidad de vida relacionada con la salud (SF-36) y la forma física. Se considerarán las variables predictoras y de confusión, como el sexo, la edad, la comorbilidad, la clase social, etc.
Análisis. Se compararán los cambios promedio observados en ambos grupos, según la intención de tratar, mediante análisis de la covarianza. Usaremos modelos de efectos mixtos para tener en cuenta la correlación intrasujeto, intramédico e intracentro.

Palabras clave: Ejercicio. Forma física. Calidad de vida. Promoción de la salud. Atención primaria. Medicina de familia. Ensayo clínico aleatorizado. Estudio multicéntrico.

PROTOCOL FOR THE MULTI-CENTRE EVALUATION OF THE EXPERIMENTAL PROGRAMME OF PROMOTION OF PHYSICAL ACTIVITY (PEPAF)

Objective. To evaluate the effectiveness of an innovative programme to promote physical activity (PEPAF) introduced into the daily consultations of the family doctor.
Design. Clinical trial with control, randomised for groups of 100 patients seen by one of the 70 doctors taking part, allocated to two parallel groups and monitored for 24 months.
Setting. 13 primary care centres coordinated through the Network of Research into Preventive and Health Promotion Activities conducted in primary care.
Participants. Sample with probability of 7000 sedentary patients, selected from among those who consulted for any reason their family doctor during the third quarter of 2003. Patients with cardiovascular disease or other problems meaning that exercise could cause adverse effects will be excluded.
Interventions. The doctors allocated to the PEPAF will design a plan of physical activity with those patients prepared to make the change. Those not prepared to will be briefly counselled and given material to help them. All will be monitored at random. The control group doctors will postpone any systematic intervention on exercise until after 2005, excepting those patients whose health problem is directly related to a sedentary life-style.
Measurements. The main measurement of results will be the increase in the level of physical activity from the base measurement to those at 3, 6, 12 and 24 months, using 7-day physical activity recall. Health-related quality of life (SF 36) and physical fitness will also be measured. Variables that might be predictive or confusing, such as sex, age, comorbidity, social class, etc., will be considered.
Analysis. The average changes observed in the two groups will be compared, on the basis of intention to treat, through analysis of covariance. We will use mixed-effect models able to cover the intra-patient, intra-doctor and intra-centre correlation.

Key words: Exercise. Physical fitness. Quality of life. Health promotion. Primary care. Family medicine. Randomised clinical trial. Multi-centre study.

[a]Epidemiólogo. [b]Doctor en Psicología. [c]Médico de familia. Unidad de Investigación de Atención Primaria de Bizkaia. Osakidetza. Servicio Vasco de Salud. Bilbao. España.

[d]Médico de Familia. Experto en Medicina Deportiva. CS Santa Bárbara. Servicio de Salud de Castilla-La Mancha. Toledo. España.

[e]Especialistas en medicina de la educación física y el deporte. Servicio de Medicina del Deporte. Diputación Foral de Bizkaia. Bilbao. España.

Correspondencia:
Gonzalo Grandes.
Unidad de Investigación de Atención Primaria-Osakidetza.
Luis Power, 18, 4ª planta.
48014 Bilbao. España.

Correo electrónico:
grandesg@ap.osakidetza.net

Proyecto financiado por el Fondo de Investigaciones Sanitarias, expediente n.º PI020015 y por la redIAPP, red de investigación acreditada y financiada por el Instituto de Salud Carlos III, exp n.º G03/170.

Relación de responsables de los equipos locales del grupo PEPAF, pertenecientes a la redIAPP (Red de Investigación en Actividades Preventivas y Promoción de la Salud): A. Ezenarro (CS Algorta), V. Salcedo (CS Galdakao) y J. Torcal (CS Basauri), por el nodo Euskadi; J. Páez (CS Camas), por el nodo Al-Andalus; M. Muñoz (CS Occidente), por el nodo Córdoba-COGRAMA; A. Estela (CS Dalt Sant Joan), por el nodo Baleares; F. Salcedo (CS Cuenca III), por el nodo Castilla la Mancha; J.A. Iglesias (CS Alamedilla) y A. Gómez Arranz (CS Casa del Barco), por el nodo Castilla León; A. Guiu (CS Serraparera), por el nodo Cataluña; L. Casariego (CS Sardoma), por el nodo Galicia; T. Gómez Gascón (CS Guayaba), por el nodo Madrid-Área 11, y G. Gil Juberias (CS Coslada), por el nodo Madrid-MADPREV.

Manuscrito recibido el 21 de julio de 2003.

Manuscrito aceptado para su publicación el 23 de julio de 2003.

Documento descargado de http://www.elsevier.es el 23/08/2010. Copia para uso personal, se prohíbe la transmisión de este documento por cualquier medio o formato.

Introducción

Son bien conocidos los beneficios para la salud que la actividad física conlleva y, por ello, se recomienda que todos los días dediquemos al menos 30 min a actividades de moderada intensidad, como caminar deprisa[1]. Lo que todavía no sabemos es cómo lograr que los pacientes sedentarios que visitan al médico de familia cumplan con esta recomendación, mediante intervenciones breves y factibles en la consulta diaria de atención primaria[2]. Posiblemente, ésta sea una de las razones por las que los médicos de familia no abordan de forma sistemática el problema del sedentarismo[3].

Muchos investigadores e instituciones dedicadas a la promoción de la actividad física han diseñado programas con este propósito, pero ninguno de los evaluados en la consulta del médico de familia ha conseguido un efecto positivo a largo plazo[2,4-8]. Si a lo anterior añadimos la alta y creciente prevalencia de sedentarismo en los países desarrollados[9,10] y la asociación con sus principales causas de morbimortalidad[1], podemos afirmar que la promoción de la actividad física en la consulta de medicina de familia es, sin duda, un área de investigación prioritaria en estos países. Además, si consideramos separadamente la actividad y la forma física, un reciente metaanálisis apunta a que es la forma física, más que la actividad física, la que mayor efecto preventivo tiene sobre la enfermedad cardiovascular[11]. Sin embargo, no existe hasta ahora ningún estudio que demuestre que el consejo médico sobre el ejercicio consigue aumentar la forma física de los pacientes.

Tras estudiar las intervenciones y programas de actividad física anteriormente aludidos, nuestro equipo de investigación ha diseñado el Programa Experimental de Promoción de la Actividad Física (PEPAF), que contiene los siguientes aspectos innovadores: implantación en un sistema de atención primaria universal y público; realización por parte de un médico de familia que accede a casi toda la comunidad y mantiene los cuidados de salud a sus pacientes de forma continua a lo largo de los años; sencillez y flexibilidad, para adaptar la intervención a la motivación del paciente para el cambio; uso de aplicaciones informáticas útiles para aconsejar y transmitir información sobre riesgos y beneficios, así como superar barreras; prescripción por escrito de un plan de actividad física, dirigido no sólo a aumentar la actividad física, sino también a mejorar la forma física de los pacientes.

Esperamos que dichos elementos innovadores hagan del PEPAF un programa efectivo y que gracias a él un importante número de pacientes sedentarios pasen a ser activos, mejoren su forma física y su calidad de vida relacionada con la salud. Este proyecto de investigación constituye la evaluación cuantitativa del logro de los resultados esperados para este programa, en las condiciones habituales de trabajo de medicina de familia.

Objetivos

Evaluar la efectividad a largo plazo del PEPAF, realizado por el médico de familia en las condiciones habituales de la consulta diaria, para mejorar el nivel de actividad física, la forma física y la calidad de vida relacionada con la salud de los usuarios de los servicios de atención primaria. Para ello, daremos respuesta de forma ordenada a las siguientes preguntas:

1. ¿Cuál es el incremento en la actividad física observado en el grupo de pacientes expuestos al PEPAF?
2. ¿Cuál es el impacto del incremento en la actividad física declarada por los pacientes sobre su forma física y calidad de vida relacionada con la salud?
3. ¿Qué parte de estas mejorías pueden ser atribuidas a la implantación del programa, en comparación con lo ocurrido con los pacientes del grupo control?
4. ¿Se mantendrá el incremento de la actividad física, la forma física y la calidad de vida producido por la intervención durante los 24 meses de seguimiento?
5. ¿Será uniforme el incremento en la actividad física, la forma física y la calidad de vida, o variará según las características de los pacientes y de los médicos? Especialmente respecto a la motivación para el cambio que presenten los pacientes a la entrada en el estudio.
6. ¿Cuál es la razón coste-efectividad y coste-utilidad del PEPAF?

Métodos

Diseño del estudio

Ensayo clínico controlado y aleatorizado por conglomerados de 100 pacientes atendidos por cada médico, que serán asignados a dos grupos paralelos: PEPAF y control (fig. 1). La asignación por médicos pretende evitar la posible contaminación del grupo control y la difícil realización simultánea de dos intervenciones diferentes en la misma consulta diaria de medicina de familia, sin alterar su dinámica habitual. Los resultados serán evaluados a largo plazo, con mediciones de la actividad física, la forma física y la calidad de vida, repetidas a cada individuo a los 3, 6, 12 y 24 meses después de la medición inicial, realizada tras su incorporación al estudio.

Emplazamiento

Cada uno de los 70 médicos de familia que colaboran en el estudio captará 100 pacientes sedentarios, seleccionados al azar, entre todos los que le consulten por cualquier motivo durante el último trimestre de 2003. Estos médicos pertenecen a 13 centros de atención primaria de los sistemas públicos de salud de 9 comunidades autónomas del Estado español. Su colaboración se ha conseguido gracias a la Red de Investigación en Actividades Preventivas y de Promoción de la Salud realizadas en Atención Primaria (http://www.rediapp.net/). En el anexo 1 se presenta la estructura del estudio.

Documento descargado de http://www.elsevier.es el 23/08/2010. Copia para uso personal, se prohíbe la transmisión de este documento por cualquier medio o formato.

FIGURA 1 Diseño del estudio.

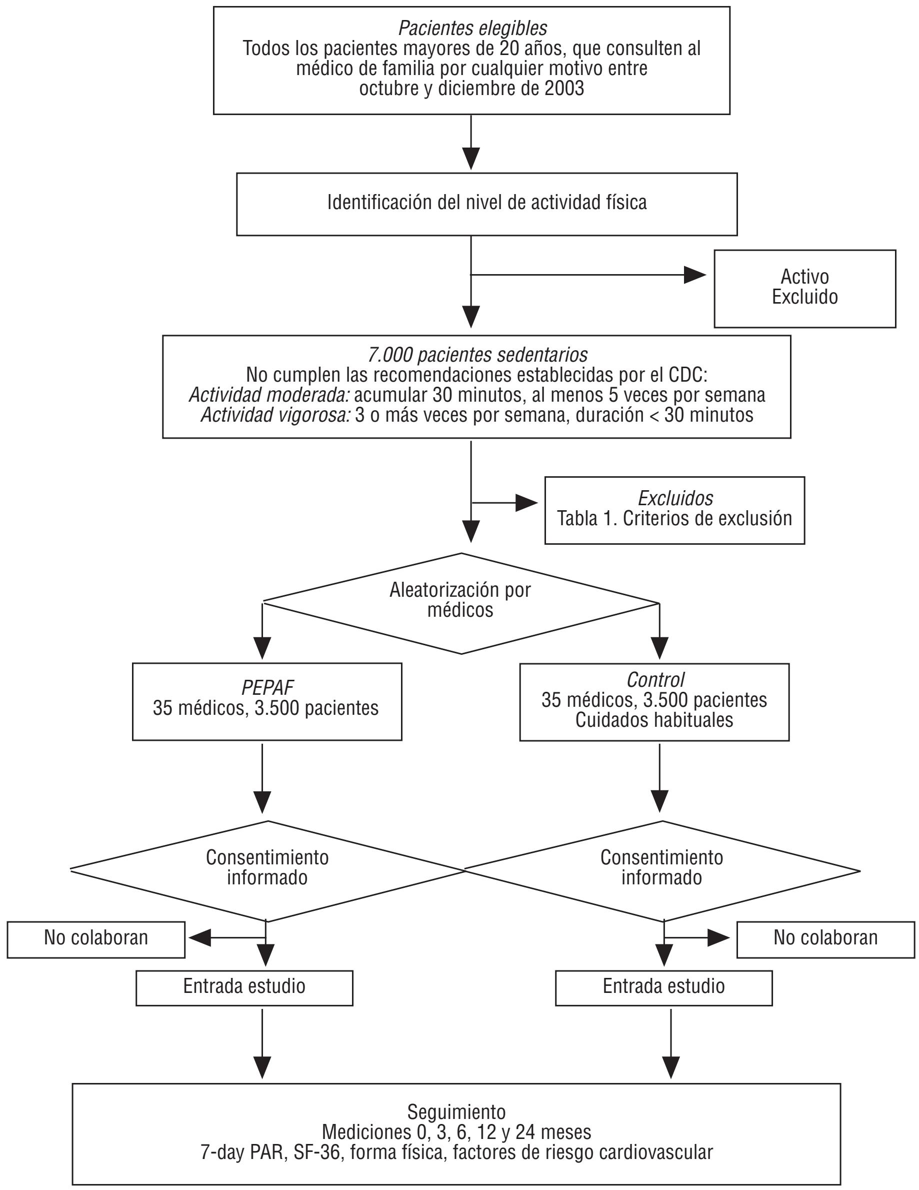

Criterios de inclusión y exclusión

Serán elegibles para el estudio todos los pacientes sedentarios, mayores de 20 años de edad, que acudan a la consulta del médico de familia por cualquier motivo, entre el 28 de octubre y el 31 de enero 2004. Se considerará paciente sedentario al que no cumpla las recomendaciones de los Centers for Disease Control and Prevention (CDC)[1]: acumular al menos 30 min de actividad física moderada 5 o más días a la semana, o realizar 3 o más sesiones a la semana de actividad intensa, con una duración mínima de 30 min cada una.

Dentro del proceso de captación de pacientes, los médicos colaboradores llevarán a cabo la verificación de los criterios de exclusión (tabla 1), a través del historial médico del paciente y la aplicación del Physical Activity Readiness Questionnaire (PAR-Q)[12].

Método de selección de los sujetos sedentarios

Durante el cuarto trimestre de 2003, se realizará una selección sistemática de 10 de los pacientes que cada médico tenga citados, uno o dos días por semana rotatorios. Una vez abordado el motivo de consulta, el médico evaluará si el paciente cumple la definición de sedentario y, en su caso, le propondrá la entrada en el estudio, mediante la presentación del consentimiento informado. Este proceso se continuará hasta que cada médico incorpore 100 pacientes sedentarios al estudio.

Cálculo del tamaño de la muestra

Una muestra de 7.000 pacientes, tras considerar un 20% de pérdidas y una correlación intraclase de 0,025, nos da un poder estadístico de al menos un 90% para detectar como significativa ($\alpha = 0,05$) una diferencia promedio de 0,47 kcal $\times$ kg^{-1} $\times$ d^{-1} en el

Documento descargado de http://www.elsevier.es el 23/08/2010. Copia para uso personal, se prohíbe la transmisión de este documento por cualquier medio o formato.

TABLA 1 Criterios de exclusión

Contraindicaciones absolutas	Contraindicaciones relativas
1. Cardiopatía isquémica	1. Presión arterial diastólica en reposo mayor de 115 mmHg o presión arterial sistólica en reposo mayor de 200 mmHg no controlada
2. Arritmia ventricular no controlada	2. Valvulopatía moderada no controlada
3. Arritmia auricular no controlada que compromete la función cardíaca	3. Alteraciones electrolíticas conocidas no controladas (hipopotasemia, hipomagnesemia)
4. Bloqueo auriculoventricular de tercer grado sin marcapasos	4. Marcapasos de frecuencia fija (utilizado raramente)
5. Insuficiencia cardíaca congestiva	5. Ectopia ventricular frecuente o compleja no controlado
6. Estenosis aórtica grave	6. Aneurisma ventricular no controlada
7. Aneurisma disecante sospechoso o conocido	7. Enfermedad metabólica descontrolada (p. ej., diabetes, tirotoxicosis o mixedema)
8. Miocarditis o pericarditis activa o sospechada	8. Enfermedad infecciosa crónica en período clínico (p. ej., mononucleosis, hepatitis, sida)
9. Tromboflebitis o trombos intracardíacos	9. Patología del aparato locomotor limitante: afección neuromuscular, musculosquelética o reumatoidea que se exacerba con el ejercicio
10. Émbolo pulmonar o sistémico reciente	10. Embarazo avanzado o complicado
11. Trasplantados renales y hepáticos	
12. Insuficiencia renal y/o hepática crónica moderada o grave	
13. EPOC grave	
14. Infecciones agudas	
15. Distrés emocional significativo (psicosis)	
16. Dificultad de contacto	
17. Activo	

gasto energético (DE = 2,055)[13,14]. Esto es así para cualquier comparación entre grupos: control e intervención o preparados y no preparados para el cambio. Esta diferencia, pequeña individualmente, supondrá un gran impacto en el conjunto de la población.

Formación de los grupos

Los médicos colaboradores serán asignados aleatoriamente y estratificados por centros, a uno de los dos grupos: PEPAF o control. De esta manera, las unidades de asignación serán grupos de 100 pacientes sedentarios por cada médico, y las unidades de observación serán dichos pacientes sedentarios. El esperado incremento en la varianza del resultado debido a la aleatorización por grupos ha sido tenido en cuenta en el cálculo del tamaño de la muestra y los análisis estadísticos. Este proceso de aleatorización lo realizará el epidemiólogo del equipo director del proyecto, de forma ciega, trabajando con los códigos de los médicos y un sistema de generación de números aleatorios del programa estadístico SAS™.

Intervención

El PEPAF, basándose en una versión reducida del modelo transteórico de las etapas de cambio[15], separa a dos tipos de pacientes: los preparados para modificar su nivel de actividad física y los no preparados. Ambos grupos de pacientes recibirán una recomendación médica asertiva personalizada, basada en las evidencias disponibles sobre los beneficios del ejercicio y los riesgos de la inactividad. Los pacientes no preparados recibirán un folleto centrado en la modificación de creencias sobre la actividad física, y de forma oportuna en consultas posteriores, volverán a ser abordados para conocer su intención de modificar su hábito sedentario. Los pacientes preparados serán emplazados a realizar una consulta adicional de menos de 20 min de duración, donde se abordarán las posibles barreras anticipadas por el paciente (falta de tiempo, recursos o problemas de salud) y se negociará un Plan de Actividad Física (PAF), centrado en el cumplimiento y superación de las recomendaciones del CDC[1]. El PAF será entregado al paciente a modo de prescripción y evaluado de forma oportunista en próximas consultas.

Los médicos del grupo control pospondrán cualquier intervención sistemática sobre el ejercicio físico hasta después de 2005, excepto con los pacientes cuyo problema de salud esté directamente relacionado con el sedentarismo.

Seguimiento de los sujetos

A los pacientes se les realizará un seguimiento durante 24 meses, dentro de los cuales tendrán lugar 5 mediciones repetidas. El seguimiento tendrá lugar en un laboratorio de mediciones, instalado en cada centro. Será responsabilidad de una enfermera contratada para el proyecto, entrenada en los diversos procedimientos de medición de las variables de estudio. Esta enfermera será ciega con respecto a la asignación de los médicos y pacientes a los grupos de intervención.

Definiciones y métodos de medida de las variables principales

El nivel de actividad física será estimado mediante el PAR[16], que proporciona una estimación del gasto energético operativizado en kilocalorías por kilogramo consumidas al día (kcal $\times$ $kg^{-1} \times d^{-1}$).

La medición de la forma física se centrará en la estimación de la resistencia cardiorrespiratoria al esfuerzo, operativizada como consumo máximo de oxígeno (VO_2max) y la capacidad de trabajo, mediante una prueba submáxima; el perímetro abdominal y el índice de masa corporal; el grosor de los pliegues grasos cutáneos (tricipital, subescapular, bicipital, pectoral, medio axilar, abdominal, suprailíaco, muslo anterior y pierna) para la determinación del porcentaje de grasa corporal, siguiendo la metodología establecida por el ACSM[17]. Asimismo, se llevará a cabo la medición de los siguientes factores de riesgo cardiovasculares y del cáncer: alcohol, tabaco, colesterol, glucemia y presión arterial.

La calidad de vida relacionada con la salud se medirá mediante la versión española del SF-36 versión 1 del Medical Outcomes Trust[18]. El SF-36 genera un perfil de ocho dimensiones de la salud y dos componentes resumen. Asimismo, utilizaremos una

Documento descargado de http://www.elsevier.es el 23/08/2010. Copia para uso personal, se prohíbe la transmisión de este documento por cualquier medio o formato.

única medida de resumen de utilidad[19], que podrá ser utilizada en el análisis económico del programa.

Además, debido a su posible efecto predictor o confusor, se tendrán en cuenta las siguientes variables: sexo, edad, nivel de estudios, clase social, diagnósticos realizados en los últimos 12 meses, fármacos crónicos y características de los médicos (sexo, edad, años de experiencia clínica, y formación previa en actividades preventivas y/o prescripción de ejercicio).

Estrategia de análisis

Todos los análisis se realizarán por intención de tratar. Los resultados de ambos grupos serán comparados utilizando modelos estadísticos de análisis de la covarianza de efectos mixtos, para tener en cuenta la correlación entre las mediciones repetidas a cada paciente y los efectos aleatorios atribuibles a pacientes y médicos[13]. Posteriormente, extenderemos dichos modelos estadísticos incluyendo las covariables hipotetizadas como posibles predictoras o confusoras, considerando además un efecto aleatorio adicional para los centros, dada la naturaleza multicéntrica del estudio. Estos análisis se realizarán con el programa estadístico SAS™.

Discusión

Es lógico que el médico de familia se plantee luchar contra la epidemia de sedentarismo, aprovechando su acceso privilegiado a la comunidad[20]. Pero hasta ahora no ha dispuesto de programas de intervención capaces de hacer activos a sus pacientes sedentarios, que resulten efectivos en las condiciones de trabajo de la consulta diaria. Si se prueba la efectividad del PEPAF, en un estudio con gran capacidad de generalización y validez en las mediciones de resultados a largo plazo, podremos dar un paso de gigante en la promoción de la actividad física desde la atención primaria. Además, este proyecto inicia una línea de trabajo e investigación sobre la actividad física como terapia especialmente apropiada en atención primaria. En este sentido, nuestro equipo ha diseñado el proyecto de investigación PReCCeS (Programa de Rehabilitación Cardíaca en el Centro de Salud) para evaluar la eficacia del ejercicio supervisado con pacientes coronarios. Este estudio se realizará a partir de 2004 dentro de la redIAPP.

Por supuesto, este protocolo presenta posibles limitaciones y amenazas. Para evitar la contaminación del grupo control, hubiera sido deseable haber realizado la asignación de los pacientes a los grupos PEPAF y control por centros, más que por médicos, pero sólo contábamos con 13 centros. Se podrían haber seleccionado entonces más centros, con un menor número de médicos en cada uno de ellos, pero el estudio se ha organizado con un mínimo de cuatro médicos por centro, para hacer rentable la inversión en laboratorios de medición de forma física y la contratación de una enfermera en cada uno de ellos para manejarlos.

La planificación del análisis por subgrupos de pacientes preparados y no preparados, así como la necesidad de detectar pequeñas diferencias individuales, importantes a nivel comunitario, obligan a trabajar con una gran cohorte de pacientes. Por tanto, se deben implantar mecanismos que minimicen las pérdidas. Una enfermera en cada uno de los centros se hará responsable del contacto periódico y el seguimiento de los pacientes. A todos los participantes se les registrarán al inicio cuatro teléfonos: el del domicilio, el móvil, el del trabajo y el de un familiar cercano; al solicitarles su consentimiento para participar en el estudio, se le explicará claramente que no es necesario que se hagan activos para seguir colaborando durante los 2 años de seguimiento.

En un estudio como éste, en el que ni médicos ni pacientes son ciegos a la intervención realizada, es muy importante que la enfermera que realiza las mediciones sí lo sea, al igual que el analista de datos. Para ello, se trabajará con un sistema de códigos que dificulte la identificación del médico y se pedirá al paciente que nunca desvele la identidad de su médico a la enfermera.

Este ensayo clínico se basa en los médicos como unidades de asignación a los grupos de comparación. Esto provoca que los pacientes de cada médico no sean independientes entre sí, lo que ha aumentado el tamaño del estudio, tres veces y media superior al de uno semejante, que se realizará con sujetos asignados independientemente a los grupos PEPAF y control. Esto obliga además a considerar la correlación entre los pacientes de cada médico, utilizando análisis más complejos que los habituales, como los modelos estadísticos de efectos mixtos. La asignación por médicos también facilita los fenómenos de confusión ligados a variables cuya distribución varíe de médico a médico, por lo que dichos modelos deberán ajustarse por posibles confusores. Una de las ventajas de estos análisis, realizados con modelos estadísticos de efectos mixtos, es que permiten aprovechar toda la información disponible, aunque sólo sea parcial en los pacientes perdidos durante el seguimiento.

Además del impacto potencial de sus resultados directos, el proyecto PEPAF aportará unas excelentes mediciones de las personas sedentarias, algunas de las cuales pasarán a ser activas durante los 2 años de seguimiento. Estas mediciones constituirán la base para posteriores estudios de cohortes que analicen el impacto del cambio en la actividad física sobre la salud.

Bibliografía

1. US Department of Health and Human Services. Physical activity and health: a report of the Surgeon General. Atlanta: US Department of Health and Human Services, Centers for Disease Control and Prevention, National Center for Chronic Disease Prevention and Health Promotion, 1996.
2. Eden KB, Orleans CT, Mulrow CD, Pender NJ, Teutsch SM. Does counseling by clinicians improve physical activity? A sum-

Documento descargado de http://www.elsevier.es el 23/08/2010. Copia para uso personal, se prohíbe la transmisión de este documento por cualquier medio o formato.

mary of the evidence for the US Preventive Services Task Force. Ann Intern Med 2002;137:208-15.
3. López de Munain J, Torcal J, López V, Garay J. Prevention in routine general practice: activity patterns and potential promoting factors. Prev Med 2001;32:13-22.
4. Pinto BM, Goldstein MG, Marcus BH. Activity counseling by primary care physicians. Prev Med 1998;27:506-13.
5. Calfas KJ, Long BJ, Sallis JF, Wooten WJ, Pratt M, Patrick K. A controlled trial of physician counseling to promote the adoption of physical activity. Prev Med 1996;25:225-33.
6. Effects of physical activity counseling in primary care. The Activity Counseling Trial: a randomnized controlled trial. JAMA 2001;286:677-87.
7. Eakin EG, Glasgow RE, Riley KM. Review of primary care-based physical activity intervention studies. J Fam Pract 2000;49:158-68.
8. Wee CC. Physical activity counseling in primary care. The challenge of effecting behavioral change. JAMA 2001;286:717-9.
9. Varo Cenarruzabeitia JJ, Martínez Gónzalez MA, Sánchez-Villegas A, Martínez Hernández JA, De Irala Estévez J, Gibney MJ. Actitudes y prácticas en actividad física: situación en España respecto al conjunto europeo. Aten Primaria 2003;31:77-86.
10. Martínez Gónzalez MA, Varo JJ, Santos JL, De Irala J, Gibney M, Kearney J, et al. Prevalence of physical activity during leisure time in the European Union. Med Sci Sports Exerc 2001;33:1142-6.
11. Williams PT. Physical fitness and activity as separate heart disease risk factors: a meta-analysis. Med Sci Sports Exerc 2001;33:754-61.
12. Thomas S, Reading J, Shephard RJ. Revision of the physical activity readiness questionnaire (PAR-Q). Can J Sport Sci 1992;17:338-45.
13. Murray DM. Design and analysis of group-randomized trials. New York: Oxford University Press, 1998.
14. Activity Counseling Research Group. Activity Counseling Trial (ACT): rationale, design, and methods. Med Sci Sports Exerc 1998;30:1097-106.
15. Prochaska JO, DiClemente CC. Stages and processes of self-change in smoking: towards an integrative model of change. J Consulting Clin Psychol 1983;51:390-5.
16. Sallis JF, Haskell WL, Wood PD. Physical activity assessment methodology in the Five-City Project. Am J Epidemiol 1985;121:91-106.
17. ACSM's Guidelines for Exercise Testing and Prescription. 6th ed. Baltimore: Lippincott Williams & Wilkins, 2000
18. Alonso J, Prieto L, Antó JM. La versión española del SF-36 Health Survey (Cuestionario de Salud SF-36): un instrumento para la medida de resultados clínicos. Med Clin (Barc) 1995;104:771-6.
19. Brazier J, Roberts J, Deverill M. The estimation of a preference-based measure of health from the SF-36. J Health Economics 2002;21:271-92.
20. Ortega Sánchez-Pinilla R, Bueno Ortiz JM, Anton Álvarez JJ, Duran Bellido E, Muñoz López J, Smithson Hodgson A, Grupo de Ejercicio Fisico de la semFYC. Ejercicio físico: una intervención poco desarrollada en atención primaria. Aten Primaria 2000;26:583-4.

ANEXO 1 Estructura de médicos colaboradores en el proyecto PEPAF

Nodo redIAPP	Responsable del equipo local	Investigadores en sus consultas
Andalucía-Al Andalus	José María Páez Pinto	M. Ángeles Tarilonte Delgado, Concepción Molina Checa, Vicente Rodríguez Pappalardo, Isabel Villafuente Fernández, Mercedes Álvarez Sanz (enfermera)
Andalucía-COGRAMA	Miguel Muñoz Álamo	Javier Montero Pérez, Jorge Martínez de la Iglesia, José Espejo Espejo, José Angel Fernández García, Cristina Aguado Taberne, José María Bueno Cobo, Antonio Yun Casalilla, Antonio González Caballero (enfermero)
Baleares-IB Salut Balears	Andreu Estela Mantolán	Txema Coll Benejam, Luis Cuixart Costa, Angels Llach Fernández, Josep M. Masuet Iglesias, Ana Moll Adan, Mónica Pons Pons y Beatriz Pons Pons (enfermeras)
Catalunya-GIAP2	Agustí Guiu Viaplana	Amadeu Díaz Prats, Xavier Martínez Artes, Oscar Solans Fernández, M. Dolores Hernández Rovira, José Ignacio Olivares Correas, Francisco Hernansanz Iglesias y Rita Ayala Mitjavila, Ana Cascos Rodríguez (enfermera) y Elena Montiel Morillo (fisioterapeuta)
Castilla la Mancha-PET-CLM	Vicente Martínez Vizcaíno	M. del Carmen García González, M. Ángeles Gabriel Escribano, M. Luscinda Velázquez Alonso, Natividad Ortega Motilla, Pedro J. Valle Sánchez Tirado y M. Jesús Segura Benito, Estela Elche Buendía (enfermera)
Castilla León-GIAP CyL	José Antonio Iglesias Valiente (Salamanca) Amparo Gómez Arranz (Valladolid)	Salamanca: José Antonio Iglesias Valiente, Manuel Gómez Marcos, Emilio Ramos Delgado, Pilar Moreno González, José Ignacio Recio Rodríguez (enfermero); Valladolid: Amparo Gómez Arranz, Miguel Ángel Díez García, Ruperto Sanz Cantalapiedra, Luis Miguel Quintero González y Nadia Carrillo Pea (enfermera)
Galicia-GIAP	Luciano Casariego Barro	Luciano Casariego Barro, Manuel Domínguez Sardiña, José Ramón Moliner de la Puente, Fernando Lago Deibe, M. Concepción Cruces Artero y M. Luisa Enríquez Velicia (enfermera)
Euskadi-UIAPB	Jesús Torcal Laguna (Basauri), Vidal Salcedo Arruti (Galdakao), Amaia Ecenarro Mugaguren (Algorta)	Basauri: Ángel Fernández Martínez, Víctor Manuel López Palacios, Begoña Etxeguren Solatxi, Víctor Landa Petralanda, Jesús Martínez Álvarez, M. Mercedes Díez López, J. Ramón Lejarza Azula, Lourdes Marijuán Arcocha; Galdakao: Vidal Salcedo Arruti, Idoia Ibáñez Aldekoa, Agurtzane Ortego Fernández, Pedro Iraguen Eguskiza, Pilar Echevarria Villega; Algorta: Amaia Ecenarro Mugaguren, M. Teresa Uribe Sánchez, Carmen Moral Fernández, Eguskiñe Iturregui San Nicolás; enfermeras: Ana Belén Fernández Alonso, Virginia Villaverde Aguirre y Judith González Pérez
Madrid-MADPREV	Gemma Gil Juberias	Gemma Gil Juberias, Lourdes Ruiz Díaz, Margarita Berzal Rosende, Agustina Criado Alcázar
Madrid-Área 11	Tomás Gómez Gascón	Javier Martínez Suberviola, José Antonio Granados Garrido, M. Ángeles Fernández Abad, M. Isabel Gutiérrez Sánchez, Carlos San Andrés Pascua, Concepción Vargas-Machuca y Cristina Díaz Pérez (enfermera)
